# A Multi-Modality Approach to the Assessment of a Right Atrium Mass in a Female Patient with Breast Cancer Undergoing Neoadjuvant Chemotherapy

**DOI:** 10.3390/diagnostics15212683

**Published:** 2025-10-23

**Authors:** Małgorzata Chlabicz, Paweł Muszyński, Joanna Kruszyńska, Piotr Kazberuk, Magdalena Róg-Makal, Magdalena Lipowicz, Urszula Matys, Anna Tomaszuk-Kazberuk, Marcin Kożuch, Sławomir Dobrzycki

**Affiliations:** 1 Department of Invasive Cardiology, Medical University of Bialystok, M. Skłodowskiej-Curie 24A, 15-276 Bialystok, Poland; 2 Department of Cardiology, Lipidology and Internal Diseases, Medical University of Bialystok, Żurawia 14, 15-569 Bialystok, Poland; 3 Faculty of Medicine, Medical University of Bialystok, Kilińskiego 1, 15-089 Białystok, Poland

**Keywords:** multi-modality, cardiac masses, echocardiography, cardio-oncology

## Abstract

Echocardiography remains a vital part of the initial assessment and monitoring of oncological patients. It allows for proper treatment selection but can also reveal life-threatening complications, including impaired left ventricular function or thromboembolism. It can rarely detect intracardiac masses that require further investigation. In the presented case, a 51-year-old female patient with left-sided breast cancer, who had undergone neoadjuvant chemotherapy, was hospitalised due to a right atrial mass identified via routine transthoracic echocardiography (TTE). Initial anticoagulation therapy showed no clinical improvement. Follow-up TTE revealed a 12 × 19 mm hyperechogenic, mobile mass in the right atrium (RA). Computed tomography angiography (CTA) ruled out pulmonary embolism and revealed that the mass was located close to the tip of the vascular access port. Transoesophageal echocardiography showed that the lesion was not connected to the vascular port. Based on location and mobility, the lesion was most consistent with a cardiac myxoma. After the Heart Team made a decision, endovascular intervention using a vacuum-assisted device was performed without complications. Histopathological examination excluded thrombosis and myxoma, revealing a fibro-inflammatory lesion. A multimodality approach is necessary to assess RA masses. However, even an extensive evaluation could be misleading, so treatment options should always be subject to the Heart Team’s decision.

**Figure 1:**
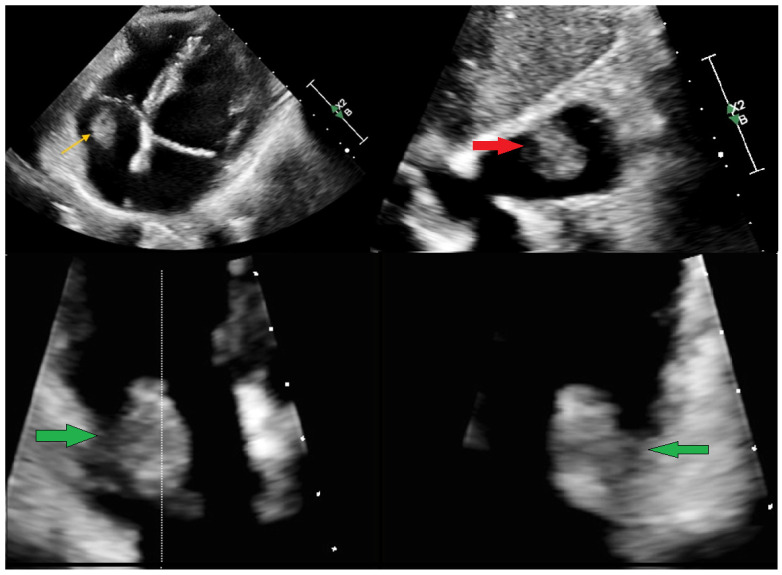
A 51-year-old female patient with a left-sided breast carcinoma after neoadjuvant cycles of chemotherapy was referred to the Emergency Department (ED) with a transthoracic echocardiography (TTE) describing an ambiguous structure located in the right atrium (RA). Initially, two months before the admission, the lesion was presumed to be of thrombotic origin. Despite prior anticoagulation therapy with nadroparin, later switched to enoxaparin, no regression of the atrial mass was observed. Referred to the ED, the patient was asymptomatic, with an unremarkable physical examination and no signs of heart failure, chest pain, syncope, or systemic inflammation. The results of routine biochemical and haematological investigations were within normal limits. The patient presented with an indwelling vascular access chemotherapy port. Repeated TTE (**above**) revealed a well-defined, hyperechoic, mobile mass of 12 × 19 mm within the RA. The lesion was attached to the atrial wall directly beneath the tricuspid valve, prolapsing through the valve orifice without evidence of flow obstruction. No abnormal echogenicity was identified around the vascular access port, and no vegetation or features suggestive of infective endocarditis were present. Global cardiac contractility was preserved, with no regional wall motion abnormalities. Yellow arrow—RA mass in apical 4-chamber view. Red arrow—RA mass in substernal 4-chamber view. Green arrow—RA mass in apical 2/4 chamber view by X-plane. The video recording can be found in Supplement S1–S3.

**Figure 2:**
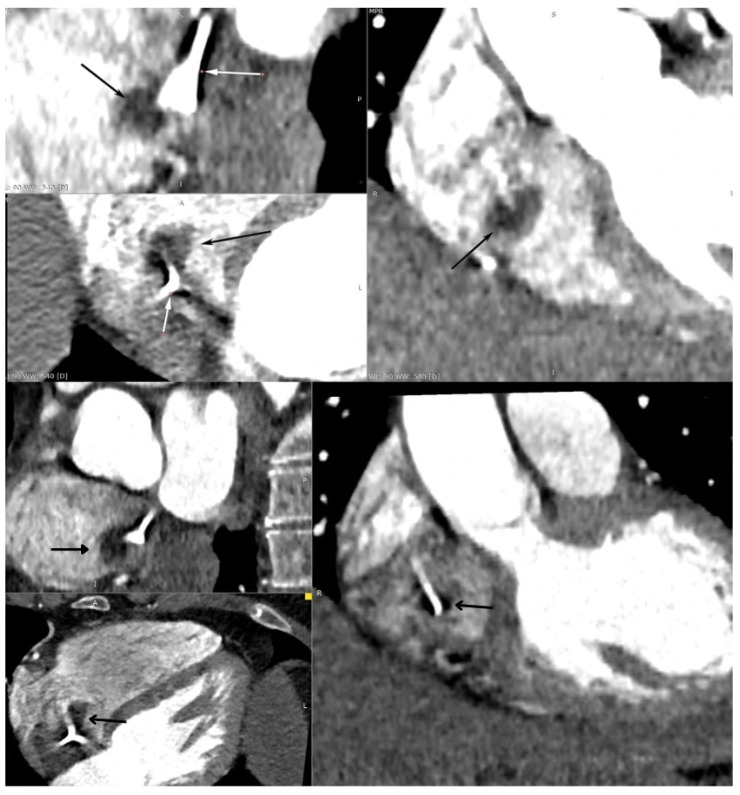
Subsequently, the patient underwent computed tomography angiography, which revealed a well-defined, oval structure measuring 15 × 20 × 18 mm located in the RA, just beneath the tip of the indwelling vascular access port. Given its proximity to the catheter tip, the possibility of an intracardiac thrombus could not be definitively excluded. White arrow—intravascular port; black arrow—RA mass. The features from CT suggesting benign character included the small size (<50 mm) and it being a single mass and well defined, with no calcifications or contrast enhancement. The only feature indicating possible malignancy was the right heart location [1,2].

**Figure 3:**
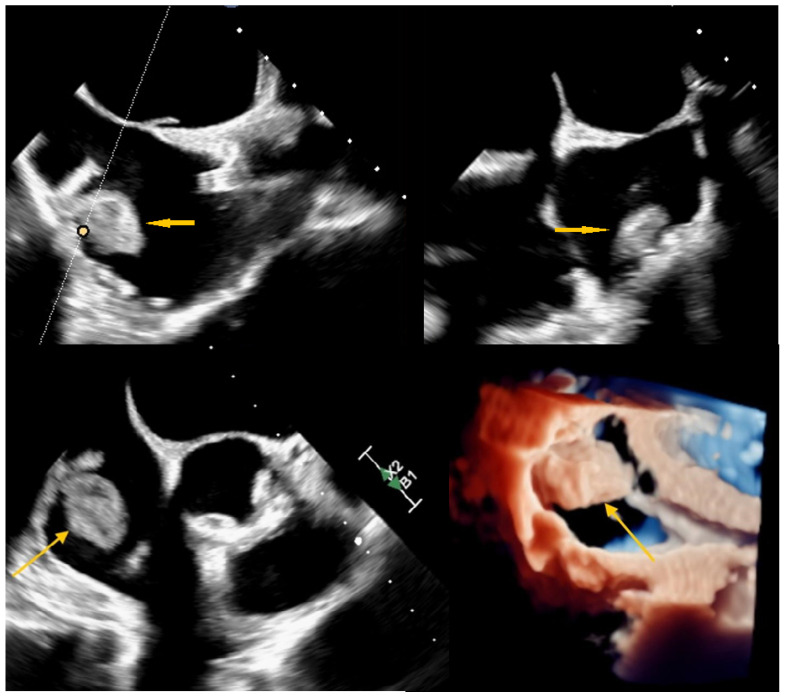
Four-chamber transoesophageal echocardiography (TEE) was subsequently performed to characterise the intracardiac mass further (yellow arrow). The study did not reveal any connection between the lesion and the vascular access port. Based on its echogenic appearance, location, and mobility, the RA mass was considered to be most consistent with a cardiac myxoma. The arrow indicates the balloting mass within the RA in 3D TEE (yellow arrow). The patient was evaluated within the framework of a multidisciplinary heart team and subsequently referred to the cardiac surgery department. A video recording can be found in Supplement S4.

**Figure 4:**
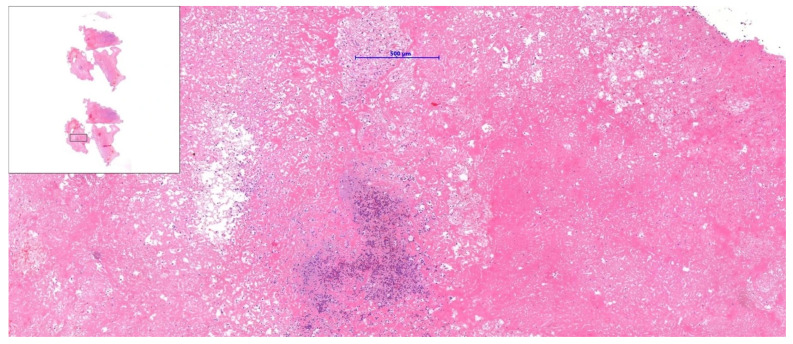
During the intervention, intracardiac material was successfully aspirated from the right atrium using a catheter-based vacuum aspiration system: 22-French (F), AngioVac (AngioDynamics, Latham, NY, USA). The procedure was performed by right (access Sheath 26-F) and left (19-F) femoral venous access, creating a veno-venous extracorporeal bypass circuit, and was guided by transoesophageal echocardiography and fluoroscopy. Subsequently, the acquired mass was submitted for histopathological evaluation. Microscopic examination demonstrated a fibro-inflammatory lesion. Initial differential considerations were excluded because there was no histological evidence of thrombus, myxoma, or other previously suspected more common pathological entities. The patient’s post-procedural course was uneventful, and she was discharged in good general condition. The patient continued her oncological treatment at the Bialystok Oncology Center, where she also underwent further echocardiographic follow-up. Subsequent evaluations showed no recurrence of the intracardiac lesion and confirmed normal cardiac function. The patient remained clinically stable, with no cardiovascular symptoms during continued cancer therapy. Regular cardiac monitoring was recommended as part of her ongoing multidisciplinary care. The case illustrates how overlapping risk factors, including malignancy, vascular access, and anticoagulation, influence diagnostic complexity. The thrombus is the most common intracardiac mass in patients with cancer; however, non-neoplastic and non-thrombotic causes should be included in differential diagnosis to avoid diagnostic bias due to cancer status [3,4]. A structured approach that incorporates advanced imaging and histological confirmation is necessary. Echocardiography remains the primary imaging tool for detecting cardiac masses; however, a multi-modality approach should be implemented, and the final decision regarding treatment should be subject to the Heart Team’s decision [3,4,5,6]. The treatment of right heart masses depends on the pre-procedure assessment of the mass by CT or cardiac magnetic resonance (CMR). CMR is considered superior to echocardiographic assessment; however, in the current case, it was not performed due to the urgency of treatment and potential risk. The potential protocol allows for distinguishing between masses and pseudomasses, including thrombi and the analysis of potential malignancy features. The estimated cumulative 5-year mortality rate for malignant tumours reaches 73% [2,7]. If malignant features are present, surgical resection with consecutive chemotherapy or radiotherapy is recommended when the initial diagnosis is confirmed by histopathology [8]. For benign masses, the approach typically depends on the presence of symptoms or the mass being large (over 10 mm). In such situations, surgical resection is preferred over conservative treatment and observation [8]. However, studies show that catheter aspiration can be an alternative, especially for patients with comorbidities and high risk, with an estimated 58.5% success rate for right heart masses [9,10]. Additionally, the study based on the MAUDE Database shows that most common complications include pulmonary embolism, perforation, arrythmia, and stroke [11].

## Supplementary Materials

The following supporting information can be downloaded at https://www.mdpi.com/article/10.3390/diagnostics15212683/s1, Video recordings of TTE and TEE are attached as Supplement S1—4-chamber apical view showing the mass in the right atrium (TTE), Supplement S2—the Xplane (2 nad 4 chamber) view showing the relation with atrium wall (TTE), Supplement S3—3-D visualisation of the mass (TTE), and Supplement S4—Xplane view (TEE).
